# Inclusion Impact of Jack Mackerel Meal in the Red Sea Bream (*Pagrus major*) Feeds Replacing Various Animal Protein Sources for Fish Meal

**DOI:** 10.1155/2024/4134106

**Published:** 2024-06-13

**Authors:** Tae Woong Kwon, Seong Il Baek, Sung Hwoan Cho

**Affiliations:** Division of Convergence on Marine Science Korea Maritime and Ocean University, Busan 49112, Republic of Korea

## Abstract

This study aims to elucidate manipulation impacts of jack mackerel meal (JMM) in the red sea bream (*Pagrus major*) feeds replacing various animal protein sources for different levels of fish meal (FM) on growth and feed availability. Two-way ANOVA experimental design was applied with three substitution sources: animal protein sources (tuna byproduct meal (TBM), chicken byproduct meal (CBM), and meat meal (MM)) and two FM substitution levels (25% and 50%). The control (Con) diet contained 60% FM. In the Con diet, 25% and 50% of FM were replaced with TBM, CBM, and MM, respectively, and then 24% jack mackerel meal (JMM) was included at the expense of FM, named as the TBM25, TBM50, CBM25, CBM50, MM25, and MM50 diets, respectively. Red sea bream juveniles averaging 11.8 g were distributed in 21 flow-through tanks (20 fish per tank) with triplicate. Fish were carefully hand-fed to apparent satiation for 8 weeks. At the end of the 8-week feeding experiment, the TBM-substituted diets produced significantly (*P* < 0.0001 for all) greater weight gain, specific growth rate (SGR), and feed consumption of fish than the CBM- and MM-substituted diets. Furthermore, dietary substitution of 25% FM achieved significantly (*P* < 0.0001, *P* < 0.0001, and *P* < 0.0003, respectively) greater weight gain, SGR, and feed consumption than dietary substitution of 50% FM based on two-way ANOVA analysis. Fish fed the TBM50 diet achieved the greatest weight gain, SGR, and feed consumption. Protein retention, biological indices, plasma and serum parameters, and the whole body chemical composition and amino acid (AA) profiles of red sea bream were not significantly influenced by dietary treatments. The TBM-substituted diets achieved significantly (*P* < 0.0001) greater economic profit index (EPI) than the CBM- and MM-substituted diets. Furthermore, dietary substitution of 25% FM achieved significantly (*P* < 0.002) greater EPI than dietary substitution of 50% FM. The TBM50 diet produced the greatest EPI. In conclusion, TBM and MM and CBM could replace 50% and 25% FM in the feeds with 24% JMM inclusion, respectively, without compromising the growth, feed utilization, plasma and serum parameters, chemical composition and AA profiles of red sea bream, and EPI. The TBM50 diet was the most desirable treatment in terms of the greatest growth performance of red sea bream and the highest economic return to farmer.

## 1. Introduction

Aquafeeds are typically more costly than feeds for other livestock species, primarily due to the high inclusion of the expensive feed ingredients, such as fish meal (FM) [1, 2]. FM is renowned as an outstanding protein source owing to its high protein content, balanced amino acid (AA) profiles, and excellent palatability and widely employed as the primary protein source in the formulation of commercially farmed fish feeds [3, 4]. However, high demand for FM in aquaculture industry worldwide and stagnant production of FM have been raising its international market price [5]. Consequently, unless the fish feed industry reduces the dependence on FM, the aquaculture industry is likely to face a critical bottleneck in the near future.

To resolve this issue, numerous protein sources have been extensively developed as a FM substitute. However, certain drawbacks of plant protein sources, such as the deficiency of some essential AA (EAA), the presence of antinutritional factors, and low nutrient digestibility, have restricted their wide use [6]. On the contrary, animal protein sources, which contain relatively high protein content with some EAA, can be considered the ideal protein replacer for FM in the formulation of the cost-effective fish feeds [1].

For instance, tuna byproduct meal (TBM) is derived from the canning process of the tuna species, such as yellowfin tuna (*Thunnus albacares*) and skipjack tuna (*Katsuwonus pelamis*) [7]. TBM is rich in crude protein (60%–64%) and lipid (8%–14%) content [8, 9] and has been widely used as a FM substitute in feeds for various fish species including olive flounder (*Paralichthys olivaceus*) [10, 11], rockfish (*Sebastes schlegelii*) [12], spotted rose snapper (*Lutjanus guttatus*) [9], and red sea bream (*Pagrus major*) [13].

Poultry byproduct meal (PBM), composed of inedible parts for humans, such as head, neck, feet, undeveloped eggs, and intestines, is one of the commonly used animal proteins in fish feeds [14, 15]. Chicken byproduct meal (CBM), a type of PBM, is notably more cost-effective than FM and provides sufficient amount of crude protein and lipid content [16, 17]. Noteworthy successes have been reported in incorporating various chicken byproducts including chicken waste meal [18], chicken offal meal [15], and CBM [17] as the substitutes for FM in the feeds of Asian seabass (*Lates calcarifer*), grass carp (*Ctenopharyngodon idella*), and olive flounder, respectively.

Meat meal (MM) is produced by rendering the inedible or unsold byproducts of livestock from slaughter operations [19]. MM has been extensively employed as an alternative source for FM in a variety of fish feeds during the past decades because of its reasonable price and abundant protein content [20, 21, 22, 23]. Especially, a pet-grade MM containing high crude protein content (≥80%) and low ash content (≤9%) has a great potential to replace for FM in fish feeds. Previous studies have demonstrated that a pet-grade MM could substitute for FM up to 40% without AA supplementation and 60% with EAA supplementation in feeds without causing any negative influence on growth of olive flounder [24, 25].

However, substitution of the excessive amount of FM with an alternative protein in feeds commonly leads to the retarded growth of fish due to the deteriorated palatability and reduced feed consumption [26, 27]. Therefore, it is advisable to incorporate a feed enhancer in formulating the low FM feeds. Incorporation of feed enhancer and/or stimulant in low FM diets has proven to be effective in elevating feed consumption and growth of fish [28, 29, 30]. In particular, Kader et al. [29] unveiled that dietary substitution of 60% FM with soy protein concentrate (SPC) led to the inferior growth of red sea bream compared to fish fed a 60% FM-based diet, but dietary substitution of 60% FM with SPC supplemented with 10% krill meal, 10% squid meal, 10% fish soluble, and their mixture achieved comparable or superior growth when juvenile red sea bream were fed with a 60% FM basal diet or one of diets substituting 60% FM with SPC without or with 10% each of krill meal, squid meal, and fish soluble (FS) and their 5% equal mixture supplementation for 50 days.

Red sea bream is a commercially important farmed fish species in Korea, along with olive flounder and rockfish. In 2023, the aquaculture production of red sea bream has gradually increased and reached 6,283 metric tons in Korea [30]. To improve aquaculture productivity of red sea bream, several researches have been conducted in nutritional aspects [26, 29, 31, 32]. In particular, our earlier study [32] unveiled that the strongest attractiveness to red sea bream was observed in jack mackerel meal (JMM), and inclusion of 40% JMM at the expense of FM in a 60% FM-based diet improved growth of red sea bream by enhancing feed intake. Likewise, a favorable effect of JMM as a feed enhancer has been reported in the diets of olive flounder [33, 34] and rockfish [35, 36]. Furthermore, jack mackerel muscle extract was also shown to be the strong feed stimulants for olive flounder [37] and Pacific bluefin tuna (*Thunnus orientalis*) [38]. The current study was, thus, carried out to evaluate the inclusion effect of JMM in the red sea bream feeds replacing FM with diverse animal protein sources on the growth, feed availability, and biochemical composition. Furthermore, the economic analysis of the study was provided.

## 2. Materials and Methods

### 2.1. Preparation of Experimental Fish and Rearing Conditions

Juvenile red sea bream were transported from a private fish farm (Tongyeong-si, Chungcheongnam-do, South Korea) and subsequently acclimated for 2 weeks in a 5-ton round flow-through tank. Fish were fed with a commercial extruded pellet (50% crude protein and 12% crude lipid) (Suhyup Feed, Uiryeong-gun, Gyeongsangnam-do, South Korea) for 2 weeks. A total of 420 juveniles (initial weight of 11.8 g) were randomly distributed into 21, 50-L rectangular flow-through tanks in triplicate of each of the seven experimental diets (20 fish/tank). Throughout the feeding trial, fish were hand-fed to apparent satiety twice daily (08:30 and 17:30) for 8 weeks. The fish were exposed to the natural photoperiod throughout the experiment. Each tank was filled with a mixture of underground seawater and sand-filtered seawater in a ratio of 1 : 1, and adequate aeration was maintained in each tank. Water quality was daily monitored using a digital multimeter (AZ-8603, AZ instrument, Taichung, Taiwan). Water temperature ranged from 20.2 to 24.1°C (22.3 ± 0.79°C; mean ± SD), dissolved oxygen ranged from 7.4 to 8.2 (7.6 ± 0.17 mg/L), salinity ranged from 31.2 to 33.6 (32.5 ± 0.53 g/L), and pH ranged from 7.1 to 7.5 (7.3 ± 0.09) during the feeding experiment. Feed consumption of each tank was recorded daily. Bottom cleaning was carried out using a siphon once a day after feeding in the morning, and dead fish were collected, weighed, and then removed on observation.

### 2.2. Preparation of the Experimental Feeds

Two-way substitution source ((TBM, CBM, and MM) × 2 substitution levels (25% and 50%)) ANOVA experimental design was adopted. Seven experimental diets (Table 1) were formulated to be isonitrogenous at 52.0% and isolipidic at 15.0% [39]. The control (Con) diet contained 60% FM and 10% fermented soybean meal as the main protein sources. In addition, the Con diet contained 17.5% wheat flour and 5% of each fish and soybean oils as the carbohydrate and lipid sources, respectively. Different levels (25% and 50%) of FM in the Con diet were replaced with TBM, CBM, and MM, respectively, and then 24% JMM was added at the expense of FM, named as the TBM25, TBM50, CBM25, CBM50, MM25, and MM50 diets, respectively. The ingredients of the experimental feeds were thoroughly blended and pelletized using a laboratory pellet extruder (Dongsung Mechanics, Busan, South Korea) equipped with a 4 mm die and then air-dried at 40°C for 48 hr. Finally, all experimental feeds were stored in a freezer at –20°C until use.

### 2.3. Sample Collection and Calculation of Growth Performance of Red Sea Bream

At the end of the feeding trial, all live fish from each tank were fasted for 24 hr, anesthetized with tricaine methanesulfonate (MS-222) at 100 ppm, and counted and weighted collectively to calculate the survival and weight gain. Ten fish from each tank were weighed and measured their total length individually. Their viscera and liver weights were measured to calculate the biological indices of fish including condition factor (K), viscerosomatic index (VSI), and hepatosomatic index (HSI). The same equations used for measurements of growth parameters, feed utilization, and biological indices of red sea bream were used [13].

### 2.4. Analysis of Plasma and Serum Parameters of Red Sea Bream

Blood samples were taken by using heparinized syringes from the caudal veins of five anesthetized red sea bream in each tank prior to dissection of fish to measure the biological indices. Plasma samples were collected after centrifugation at 2,700x *g* at 4°C for 10 min and stored in a freezer at –70°C as separate aliquots for analysis of aspartate aminotransferase (AST), alanine aminotransferase (ALT), alkaline phosphatase (ALP), total bilirubin (T-BIL), total cholesterol (T-CHO), triglyceride (TG), total protein (TP), and albumin (ALB). These plasma parameters were measured using an automatic chemistry system (Fuji Dri-Chem NX500i, Fujifilm, Tokyo, Japan) with the following Fuji Dri-Chem Slides: GOT/AST-PIII (Catalog No. 15809542, Fujifilm, Tokyo, Japan), GPT/ALT-PIII (Catalog No. 16654035), ALP-PIII (Catalog No. 16653964), TBIL-PIII (Catalog No. 16654061), TCHO-PIII (Catalog No. 16654073), TG-PIII (Catalog No. 16654085), TP-PIII (Catalog No. 16654097), and ALB-P (Catalog No. 16653952), respectively. Blood samples were taken by using syringes from the caudal veins of five anesthetized red sea bream in each tank prior to dissection of fish. Serum samples were collected after centrifugation at 2,700x *g* at 4°C for 10 min and stored in a freezer at –70°C. Superoxide dismutase (SOD) was measured by competitive inhibition enzyme immunoassay technique using a fish SOD ELISA kit (Catalog No. MBS705758, Mybiosource Inc., San Diego, CA, USA) following the standard protocol of the manufacturer. The absorbance values were measured at 450 nm using a microplate reader (Infinite M Plex, Tecan, Männedorf, Switzerland) and then used to calculate SOD activity. The turbidimetric assay for lysozyme was performed according to Lange et al. [40]. In brief, test serum (100 *µ*L) was added to 1.9 mL suspension of *Micrococcus lysodeikticus* (0.2 mg/mL; Sigma, St. Louis, MO, USA) in a 0.05 M sodium phosphate buffer (pH 6.2). The reactions were performed at 25°C, and absorbance at 530 nm was recorded between 0 and 60 min on a spectrophotometer. The amount of enzyme required to produce a 0.001/min reduction in absorbance was regarded as the lysozyme activity unit.

### 2.5. Analysis of the Biochemical Composition of the Experimental Feeds and Fish

Ten fish at the beginning of the feeding experiment and all remaining red sea bream (≥7) from each tank at the completion of the feeding experiment were sampled for the biochemical analysis of the whole body. The chemical composition analyses were determined following the standard procedure [41].

All AA (except for methionine, cysteine, and tryptophan) in the main protein sources, experimental feeds, and whole body of fish were analyzed using an AA analyzer (L-8800 Auto-analyzer: Hitachi, Tokyo, Japan) via ion-exchange chromatography after hydrolyzing with 6 N HCl at 110°C for 24 hr. For methionine and cysteine analysis, samples underwent performic acid oxidation for 24 hr at a temperature below 5°C to produce the methionine sulfone and cysteic acid. All fatty acids (FA) in the main protein sources, experimental feeds, and whole body of fish were extracted by the mixture of chloroform and methanol (2 : 1 v/v) according to the method of Folch et al. [42], and FA methyl esters were prepared by transesterification with 14% BF_3_-MeOH (Sigma, St. Louis, MO, USA). The same methods and procedures of the biochemical composition of the samples were used [13].

### 2.6. Analysis of the Economic Parameters of the Study

The economic parameters were conducted using USD as the currency type. The economic conversion ratio (ECR) and economic profit index (EPI) were calculated following the methods outlined by Bicudo et al. [43] and Montenegro et al. [44]. The prices of feed ingredients and fish were determined using the exchange rate USD 1 = 1,315 won (South Korean currency). The price of red sea bream was estimated at 20.29 USD/kg. The price of the experimental feeds was estimated by multiplying the individual cost of feed ingredient by their respective cost per kg and summing the values obtained from all the ingredients in the experimental feeds. In reference to the market prices of feed ingredients in Korea, the price (USD/kg) of each ingredient was as follows: FM = 2.02; JMM = 2.37; TBM = 1.22; CBM = 0.88; MM = 1.16; fermented soybean meal = 0.66; wheat flour = 0.52; fish oil = 2.59; soybean oil = 1.68; vitamin premix = 7.76; mineral premix = 6.24; and choline = 1.22.

### 2.7. Statistical Analysis

All data were analyzed using SPSS version 24.0 (SPSS Inc., Chicago, IL, USA). All percentage data were arcsine-transformed before statistical analysis. Data were evaluated for normality and homogeneity of variance, using Shapiro–Wilk and Levene tests, respectively, and there were no violations (*P* > 0.05). Two-way ANOVA was used to analyze the effects of FM substitution source, substitution level, and their interaction, at a significance level of *P* < 0.05. One-way ANOVA was also used to compare the means of dietary treatments including the Con diet. When significant differences (*P* < 0.05) were detected among dietary treatments, those data were tested by Tukey's honestly significant difference post hoc test.

## 3. Results

### 3.1. Amino and Fatty Acid Profiles of the Experimental Feeds

All EAA contents in TBM, CBM, and MM, except for histidine and tryptophan, arginine and tryptophan, and arginine and histidine, respectively, were relatively lower than those of FM (Table 2). However, histidine, isoleucine, tryptophan, and valine contents in JMM were relatively higher than those of FM. All experimental feeds satisfied the dietary requirements of arginine (2.37% of the diet), lysine (1.79% of the diet), and valine (0.90% of the diet) for red sea bream.

Total content of saturated FA (∑SFA) in TBM and MM was relatively higher than that in FM, while the total content of monounsaturated FA (∑MUFA) in JMM, TBM, CBM, and MM was relatively higher than that in FM (Table 3). The total content of n-3 highly unsaturated FA (∑n-3 HUFA) including eicosapentaenoic acid (EPA, C20 : 5n-3) and docosahexaenoic acid (DHA, C22 : 6n-3) of TBM, CBM, and MM was lower than that of FM. The content of EPA and DHA and ∑n-3 HUFA of JMM was higher than those of FM. Increased FM substitution levels with TBM, CBM, and MM in diets with JMM inclusion led to increased ∑SFA and ∑MUFA but decreased the content of EPA and DHA and ∑n-3 HUFA.

### 3.2. Survival and Growth Performance of Red Sea Bream

**Figure 1:**
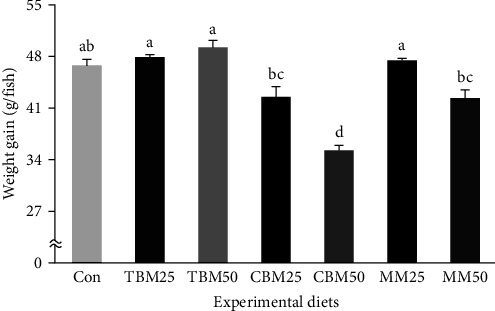
Weight gain (g/fish) of red sea bream (*Pagrus major*) fed the experimental diets for 8 weeks (means of triplicate) (substitution source, *P* < 0.0001; substitution level, *P* < 0.0001; and interaction, *P* < 0.003). Significant differences in dietary treatments were obtained from Tukey's honestly significant differences post hoc test (*P* < 0.0001).

**Figure 2:**
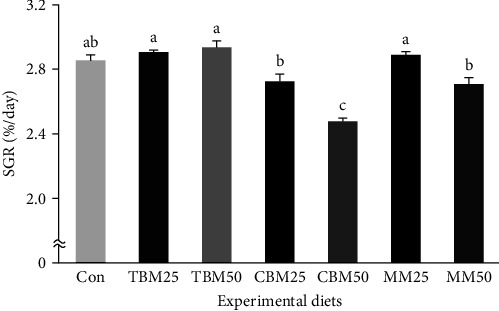
Specific growth rate (SGR (%/day)) of red sea bream (*Pagrus major*) fed the experimental diets for 8 weeks (means of triplicate) (substitution source, *P* < 0.0001; substitution level, *P* < 0.0001; and interaction, *P* < 0.005). Significant differences in dietary treatments were obtained from Tukey's honestly significant differences post hoc test (*P* < 0.0001).

Survival of red sea bream ranged from 90.0% to 98.3%, but it was not significantly changed by either substitution source (*P* > 0.2) or substitution level (*P* > 0.3) (Table 4). However, the TBM-substituted diets (48.6 g/fish and 2.92%/day) achieved significantly (*P* < 0.0001 for both) greater weight gain and SGR of red sea bream than the MM-substituted diets (44.9 g/fish and 2.80%/day), which also achieved significantly greater weight gain and SGR than the CBM-substituted diets (38.9 g/fish and 2.60%/day, respectively). Furthermore, dietary substitution of 25% FM (45.9 g/fish and 2.84%/day) produced significantly (*P* < 0.0001 for both) greater weight gain and SGR compared to dietary substitution of 50% FM (42.3 g/fish and 2.71%/day, respectively). Their significant interactions (*P* < 0.003 and *P* < 0.005, respectively) on weight gain and SGR of red sea bream were also observed. Weight gain and SGR of red sea bream fed the TBM25, TBM50, and MM25 diets were superior to red sea bream fed the CBM25, CBM50, and MM50 diets (*P* < 0.0001 for both) but comparable to red sea bream fed the Con diet (Figures 1 and 2).

### 3.3. Feed Availability and Biological Indices of Red Sea Bream

The TBM-replaced diets (46.6 g/fish) achieved significantly (*P* < 0.0001) higher feed consumption than the MM-replaced diets (44.0 g/fish), which also achieved significantly (*P* < 0.05) higher feed consumption than the CBM-replaced diets (38.4 g/fish). Furthermore, dietary replacement of 25% FM (44.1 g/fish) achieved significantly (*P* < 0.003) higher feed consumption than the dietary replacement of 50% FM (42.0 g/fish). Their significant interaction (*P* < 0.003) on feed consumption of fish was also observed. Feed consumption of fish fed the TBM25 and TBM50 diets were significantly (*P* < 0.0001) higher than that of red sea bream fed the CBM25, CBM50, and MM50 diets but not significantly (*P* > 0.05) different from that of fish fed the Con and MM25 diets.

Dietary substitution of 25% FM (1.05 and 2.03) produced significantly (*P* < 0.005 and *P* < 0.0001, respectively) higher FE and PER than dietary substitution of 50% FM (1.01 and 1.94, respectively). FE of red sea bream fed the Con diet was significantly (*P* < 0.004) higher than that of red sea bream fed the CBM50 and MM50 diets but not significantly (*P* > 0.05) different from that of red sea bream fed the TBM25, TBM50, CBM25, and MM25 diets. PER of red sea bream fed the TBM25, CBM25, and MM25 diets was significantly (*P* < 0.002) higher than that of fish fed the CBM50 and MM50 diets but not significantly (*P* > 0.05) different from that of fish fed the Con and TBM50 diets. However, PR of red sea bream was not significantly changed by either substitution source (*P* > 0.6) or substitution level (*P* > 0.1).

Biological indices of red sea bream, such as K (2.05–2.11 g/cm^3^), VSI (7.18%–7.72%), and HSI (2.08%–2.30%), were not significantly influenced by either substitution source (*P* > 0.6, *P* > 0.5, and *P* > 0.6, respectively) or substitution level (*P* > 0.3, *P* > 0.9, and *P* > 0.9, respectively).

### 3.4. Plasma and Serum Parameters of Red Sea Bream

None of plasma AST (50.3–55.3 U/L), ALT (8.2–9.2 U/L), ALP (167.0–182.7 U/L), T-BIL (0.8–0.9 mg/dL), T-CHO (246.4–264.0 mg/dL), TG (381.1–393.7 mg/dL), TP (4.7–5.1 g/dL), and ALB (1.1 g/dL) of red sea bream was significantly changed by either substitution source (*P* > 0.05 for all) or substitution level (*P* > 0.05 for all) (Table 5).

Serum lysozyme activity (96.7–125.0 U/mL) and SOD (67.0%–67.7%) of red sea bream was not significantly influenced by either substitution source (*P* > 0.3 and *P* > 0.7, respectively) or substitution level (*P* > 0.1 and *P* > 0.9, respectively).

### 3.5. Biochemical Composition of the Whole Body of Fish

The whole-body moisture (64.2%–68.2%), crude protein (17.6%–18.1%), crude lipid (9.2%–10.9%), and ash (4.6%–5.2%) content of red sea bream were not significantly influenced by either substitution source (*P* > 0.8, *P* > 0.9, *P* > 0.4, and *P* > 0.9, respectively) or substitution level (*P* > 0.7, *P* > 0.6, *P* > 0.3, and *P* > 0.6, respectively) (Table 6).

The AA profiles of the whole-body red sea bream were not significantly changed by either substitution source (*P* > 0.05 for all) or substitution level (*P* > 0.05 for all) (Table 7).

Dietary substitution of 50% of FM (34.76% of wet weight) produced significantly (*P* < 0.01) higher ∑MUFA of the whole-body red sea bream than dietary substitution of 25% of FM (33.67% of wet weight) (Table 8). The ∑MUFA of the whole-body red sea bream fed the TBM50, CBM50, and MM50 diets was significantly (*P* < 0.003) higher than that of red sea bream fed the Con diet but not significantly (*P* > 0.05) different from that of red sea bream fed the TBM25, CBM25, and MM25 diets.

The TBM-replaced diets (6.86% of wet weight) produced significantly (*P* < 0.0001 for all) higher ∑n-3 HUFA of the whole-body fish than the CBM- (5.93% of wet weight) and MM-replaced diets (5.97% of wet weight). Furthermore, dietary replacement of 25% FM (6.60% of wet weight) produced significantly (*P* < 0.0001 for all) higher ∑n-3 HUFA of fish than dietary replacement of 50% FM (5.92% of wet weight). Their significant interaction (*P* < 0.01) on ∑n-3 HUFA of whole body fish was also observed. The EPA, DHA, and ∑n-3 HUFA of the whole body of red sea bream fed the Con diet were significantly (*P* < 0.0001 for all) higher than those of red sea bream fed all other diets, except for the EPA of fish fed the TBM25 diet.

### 3.6. Economic Parameters of the Study

The price of the Con diet (1.73 USD/kg) was the highest, and diet price decreased with dietary increased FM substitution levels with all animal proteins (Table 9). The TBM-replaced diets (1.28 and 1.15 USD/fish) achieved significantly (*P* < 0.0001 for both) higher ECR and EPI than the MM-replaced diets (1.24 and 1.08 USD/fish), which also achieved significantly higher ECR and EPI than the CBM-replaced diets (1.17 and 0.97 USD/fish, respectively). Furthermore, dietary substitution of 25% FM (1.27 and 1.10 USD/fish) achieved significantly (*P* < 0.0001 and *P* < 0.002, respectively) higher ECR and EPI than dietary substitution of 50% FM (1.19 and 1.03 USD/fish, respectively). Their significant interaction (*P* < 0.002) on EPI was also observed. ECR of the Con diet was significantly (*P* < 0.0001) higher than that of all other experimental diets, except for the TBM25 diet. EPI of the TBM25, TBM50, and MM25 diets was significantly (*P* < 0.0001) higher than that of the CBM25, CBM50, and MM50 diets, but not significantly (*P* > 0.05) different from that of the Con diet. The greatest EPI (1.16 USD/fish) was obtained in the TBM50 diet.

## 4. Discussion

The TBM-substituted diets led to superior weight gain, SGR, and feed intake of red sea bream to the CBM- and MM-substituted diets as long as 24% JMM was included instead of FM in the low FM diets in this experiment. These results indicated that TBM could serve as a more effective substitute for FM than CBM and MM in the red sea bream feeds. Likewise, Kim et al. [45] reported that TBM is a more suitable replacer for FM than various animal protein sources (CBM, hydrolyzed chicken offal meal, meat and bone meal, MM, and blood meal) in the olive flounder feeds. Furthermore, FM up to 50% could be replaced with TBM in the feeds of olive flounder [10] and rockfish [12] and 75% in the feed of spotted rose snapper [9] without causing any adverse impacts on the growth performance and feed consumption.

Dietary substitution of 25% FM outperformed in weight gain, SGR, and feed intake of red sea bream over dietary substitution of 50% FM in this experiment. Similarly, increased FM substitution levels with various animal proteins in diets deteriorated feed intake and growth of fish [25, 46, 47, 48, 49]. Substitutability of FM with animal proteins in fish feeds is commonly constrained by low feed intake due to deteriorated palatability and acceptability of low FM diets by fish [26, 27].

Red sea bream fed the TBM25 and TBM50 diets exhibited a slight, but not significant, improvement in weight gain, SGR, and feed intake compared to red sea bream fed the Con diet, suggesting that substitution of FM up to 50% with TBM in diets with 24% JMM inclusion has the favorable effects on feed consumption and growth performance. Baek and Cho [13] proved that FM up to 40% in diet could be substituted with TBM without retardation of the growth and feed consumption of red sea bream. In considering the results of this study and Baek and Cho [13], we can conclude that the substitutability of FM with TBM in the red sea bream diets increased from 40% to 50% when 24% JMM instead of FM was included in diets. Furthermore, slight but not significant improvement in weight gain and SGR of red sea bream fed the CBM25 diet compared to red sea bream fed the Con diet also indicated that 25% FM could be substituted with CBM in the red sea bream feed without compromising growth performance as long as 24% JMM instead of FM was included in diet. In addition, no discernible differences in weight gain, SGR, and feed consumption of red sea bream fed the MM50 diet compared to red sea bream fed the Con diet might imply that FM up to 50% could be substitutable with MM in the red sea bream feed without compromising growth performance and feed consumption as long as 24% JMM was included in diet. Nevertheless, the MM25 diet achieved superior weight gain and SGR compared to fish fed the MM50 diet in this study. Although MM was an economically valuable replacer for FM in fish feeds, its substitutability would be still limited to some extent in fish feeds [21, 50]. FM up to 40% [24] and 60% with EAA supplementation [25] could be substitutable with MM in the olive flounder feeds without any unfavorable effect on growth performance. Similarly, Gunathilaka et al. [47] also unveiled that substitution of 50% FM (a mixture of TBM, pollock meal, and sardine meal at a ratio of 2 : 1 : 1) with MM in the red sea bream diets achieved comparable growth to fish fed the 60% FM-based feed.

Dietary replacement of the excessive amount of FM with an alternative protein commonly led to the deteriorated palatability and lowered feed consumption of red sea bream and gilthead seabream (*Sparus aurata*) [26, 27], eventually limiting its substitutability for FM in diets. No discernible difference in feed consumption of red sea bream fed the Con, TBM25, TBM50, MM25, and MM50 diets in the present study probably indicated that manipulation of 24% JMM in the low FM diets replacing 25% and 50% FM with either TBM or MM exhibited a feed enhancer effect on red sea bream.

One of the limiting factors to determine substitutability of an alternative source for FM in fish feeds is the imbalanced EAA content [29, 51]. Gasco et al. [52] stressed that reduced growth performance of fish fed low FM diets substituting high levels of FM with PBM is often associated with deficiency in EAA content. Requirements of arginine (2.37% in the diet) [53], lysine (1.79% in the diet) [54], and valine (0.90% in the diet) [55] for growth of red sea bream were fulfilled in all experimental feeds in this study. Therefore, the variation in EAA content in the experimental feeds might not have a negative effect on the growth of red sea bream.

In diets of marine fish, n-3 HUFA, such as EPA and DHA, is typically considered nondispensable FA for stable growth and survival of fish [56]. Dietary EPA and DHA requirements for juvenile red sea bream were estimated to be 1% (6.67% of total FA in this study) and 0.5% (3.33% of total FA in this study), respectively, especially when DHA and EPA, respectively, in diets were not present [57]. In their study, however, a diet supplemented with both 0.25% EPA and 0.25% DHA (1.67% of total FA for each in this study) produced comparable growth to red sea bream fed diets including 1% EPA or 0.5% DHA. The content of EPA (1.90%–3.87% of total FA) and DHA (2.38%–5.01% of total FA) in all experimental feeds in the present study appeared to fulfill their dietary requirements for red sea bream. However, the DHA:EPA ratio in the experimental diets ranged from 0.97 to 1.60. Previous studies have underscored the pivotal role of dietary DHA:EPA ratio in influencing the growth of carnivorous fish species, and adequate dietary DHA:EPA ratios for golden pompano (*Trachinotus ovatus*) [58] and starry flounder (*Platichthys stellatus*) [59] were estimated to be 1.40 and 1.24, respectively. Although an optimal DHA:EPA ratio in diet varied among fish species [60] and it was not known for red sea bream, the lowest DHA:EPA ratio in the CBM50 diet might seem to partially contribute to the poorest growth performance of red sea bream in considering the fact that the efficiency of DHA is twice as high as that of EPA [57].

Dietary replacement of 25% FM led to superior FE and PER of red sea bream to dietary replacement of 50% FM in this experiment, being consistent with other studies reporting that FE and PER of fish tended to decrease with increased dietary FM substitution levels with PBM and MM [25, 47, 61]. Deteriorated feed utilization of fish might be resulted from poor digestibility and deficiencies in some EAA in the alternative animal proteins for FM in low FM feeds [1, 51].

Fish fed the TBM25, TBM50, CBM25, and MM25 diets led to comparable FE and PER to fish fed the Con diets. Similarly, replacement of FM up to 50% and 75% with TBM in the diets of olive flounder [10] and rockfish [12], respectively, did not cause any negative impact on feed utilization. However, inferior FE of red sea bream fed the CBM50 and MM50 diets, but comparable FE of red sea bream fed the TBM50 diet to fish fed the Con diet indicated that red sea bream could effectively utilize TBM rather than CBM and MM as a replacer for FM when 50% FM were replaced with TBM, CBM, and MM in diets with JMM inclusion. Likewise, olive flounder fed the TBM-replaced diets achieved superior FE to fish fed the CBM- and MM-replaced diets when 30% FM were replaced with TBM, CBM, and MM in diets [45]. Substitutability of animal protein sources for FM in fish diets highly varied depending on different grades, manufacturing process, and proportion of less digestible materials in animal proteins [51]. The PR and biological indices (K, VSI, and HSI) of red sea bream were not altered by dietary treatments. Likewise, substitution of various animal protein sources for FM in the feeds of red sea bream [47, 62], gilthead sea bream [63], European seabass (*Dicentrarchus labrax*) [64], and Atlantic salmon (*Salmo salar*) [65] did not alter the biological indices.

Blood parameters of fish are useful for the diagnosis of diseases and assessment of the extent of damage to the blood, serving as the effective indicator to evaluate the health status of various fish species [66]. Plasma parameters can be considered as the indicators of physiological stress to environment and health status of fish [47, 67]. Serum parameters, lysozyme functions as a bacteriolytic enzyme, widely distribute throughout the body as an integral component of the innate defense mechanisms in most animals [68], and SOD acts as an antioxidant enzyme, protecting cells from damage caused by oxidative activities [69]. Neither substitution source nor substitution level in diets exhibited remarkable effects on the plasma and serum parameters of red sea bream in the present experiment, probably demonstrating that substitution of 25% and 50% FM with various animal protein sources in diets with JMM inclusion did not influence the health status of red sea bream. Likewise, dietary substitution of 50% FM with CBM and MM did not alter the plasma and serum parameters of red sea bream in the 12-week feeding experiment [47]. Aya et al. [70] reported that the plasma parameters as well as serum lysozyme and myeloperoxidase activities of olive flounder were not affected by dietary FM replacement by fermented TBM.

The chemical composition and AA profiles of the whole-body red sea bream were unaffected by either dietary substitution source or substitution level in the present study. Similar results showing that the chemical composition and AA profiles of whole-body fish were not affected by dietary FM replacement with various animal protein sources [47, 66, 71]. Higher content of EPA, DHA, and ∑n-3 HUFA in the whole body was found in red sea bream fed the Con diet compared to fish fed all other experimental diets, except for the EPA content of fish fed the TBM25 diet. This was well reflected from lower EPA, DHA, and ∑n-3 HUFA content in the 50% FM-replaced diets than the 25% FM-replaced diets. Similarly, the FA profiles of the whole body of fish were altered by dietary FM replacement with animal protein sources [45, 66, 72].

Feed cost typically accounts for 40%–70% of the total cost of aquaculture production [6, 73]. Therefore, the fundamental objective of feeding practices is lowering feed cost, but enhancing the economic return to fish farmer. In terms of economic view, the EPI serves as a suitable parameter to assess the profitability of feed for farmers, as it takes into account factors such as weight gain, feed consumption, feed cost, and the selling price of fish. The EPI of fish feeds can be optimized by substituting fish meal with cost-effective protein sources at suitable levels [27, 44, 74, 75]. Even though the price of TBM was relatively higher than that of CBM and MM, the TBM-replaced diets achieved remarkably greater EPI than CBM- and MM-replaced diets in this study. Byproduct meal from fisheries including TBM is considered not only a viable replacement for FM, but also a significantly economical and sustainable protein source [12, 76]. Furthermore, dietary replacement of 25% FM with animal protein sources led to the greater EPI than dietary replacement of 50% FM in the current experiment. This was reflected from superior weight gain of fish receiving the former than the latter. The greatest EPI and the greatest weight gain were achieved in red sea bream fed the TBM50 diet, implying that replacement of 50% FM with TBM in diet with JMM inclusion could bring about the greatest economic return to farmer.

In conclusion, FM up to 50% and 25% could be substitutable with TBM and MM and CBM, respectively, in the red sea bream feeds with 24% JMM inclusion without compromising growth performance, feed availability, biological indices, plasma and serum parameters, chemical composition, and AA profiles. Moreover, the greatest growth performance and the highest could be achieved in fish fed the TBM50 diet.

## Figures and Tables

**Table 1:** Ingredient and chemical composition of the experimental diets (%, DM basis).

	Experimental diets						
	Con	TBM25	TBM50	CBM25	CBM50	MM25	MM50
Ingredient (%)							
Fish meal (FM)^1^	60	21	6	21	6	21	6
Jack mackerel meal (JMM)^2^	—	24	24	24	24	24	24
Tuna byproduct meal (TBM)^3^	—	18.5	37	—	—	—	—
Chicken byproduct meal (CBM)^4^	—	—	—	17.8	35.6	—	—
Meat meal (MM)^5^	—	—	—	—	—	12.7	25.4
Fermented soybean meal	10	10	10	10	10	10	10
Wheat flour	17.5	15.1	12.2	16.5	14.9	20.3	22.7
Fish oil	5	5	5	5	5	5	5
Soybean oil	5	3.9	3.3	3.2	2.0	4.5	4.4
Vitamin premix^6^	1	1	1	1	1	1	1
Mineral premix^7^	1	1	1	1	1	1	1
Choline	0.5	0.5	0.5	0.5	0.5	0.5	0.5
Nutrients (%)							
Dry matter	95.8	95.7	95.6	95.6	95.3	95.4	95.6
Crude protein	52.2	51.6	51.9	52.2	52.2	51.6	52.0
Crude lipid	14.9	14.6	14.7	15.3	14.7	15.3	14.7
Ash	10.1	11.8	13.4	11.0	12.4	9.6	7.9

^1^Fish meal (FM) (crude protein, 71.8%; crude lipid, 8.0%; and ash, 16.1%) (the mixture of sardine meal and anchovy meal at the ratio of 1 : 1) imported from Chile (USD 2.02/kg FM, USD 1 = 1,315 won (Korean currency)). ^2^Jack mackerel meal (JMM) (crude protein, 72.8%; crude lipid, 9.8%; and ash, 15.4%) imported from Chile (USD 2.37/kg). ^3^Tuna byproduct meal (TBM) (crude protein, 63.5%; crude lipid, 9.8%; and ash, 18.62%) was purchased from Woojinfeed Ind. Co., Ltd. (Incheon, Korea) (USD 1.22/kg). ^4^Chicken byproduct meal (CBM) (crude protein, 65.0%; crude lipid, 13.6%; and ash, 16.4%) was purchased from Chamfre Co., Ltd. (Buan-gun, Jeollabuk-do, Korea) (USD 0.88/kg). ^5^Meat meal (MM) (crude protein, 85.6%; crude lipid, 8.5%; and ash, 5.9%) was purchased from Daekyun Oil and Transportation Co., Ltd. (Busan, Korea) (USD 1.16/kg). ^6^Vitamin premix contained the following amount which were diluted in cellulose (g/kg mix): L-ascorbic acid, 121.2; DL-*α*-tocopheryl acetate, 18.8; thiamine hydrochloride, 2.7; riboflavin, 9.1; pyridoxine hydrochloride, 1.8; niacin, 36.4; Ca-D-pantothenate, 12.7; myo-inositol, 181.8; D-biotin, 0.27; folic acid, 0.68; p-aminobenzoic acid, 18.2; menadione, 1.8; retinyl acetate, 0.73; cholecalciferol, 0.003; and cyanocobalamin, 0.003. ^7^Mineral premix contained the following ingredients (g/kg mix): MgSO_4_·7H_2_O, 80.0; NaH_2_PO_4_·2H_2_O, 370.0; KCl, 130.0; ferric citrate, 40.0; ZnSO_4_·7H_2_O, 20.0; Ca-lactate, 356.5; CuCl, 0.2; AlCl_3_·6H_2_O, 0.15; KI, 0.15; Na_2_Se_2_O_3_, 0.01; MnSO_4_·H_2_O, 2.0; and CoCl_2_·6H_2_O, 1.0.

**Table 2:** Amino acid profiles (% of the diet) of the protein sources and experimental diets.

	Protein sources					Requirement	Experimental diets						
	FM	JMM	TBM	CBM	MM		Con	TBM25	TBM50	CBM25	CBM50	MM25	MM50
Essential amino acid (%)													
Arginine	4.02	3.96	3.53	4.14	5.71	2.37^3^	2.94	2.77	2.68	3.10	3.18	3.37	3.63
Histidine	1.61	2.26	1.88	1.29	1.61	—	1.21	1.41	1.49	1.27	1.16	1.27	1.15
Isoleucine	2.48	2.75	2.41	1.95	1.99	—	2.21	2.28	2.38	2.20	2.12	2.15	2.02
Leucine	5.23	4.86	4.35	4.08	4.42	—	3.64	3.58	3.45	3.39	3.27	3.26	3.05
Lysine	5.53	5.28	4.54	3.92	4.13	1.79^4^	3.75	3.69	3.56	3.31	3.12	3.14	2.89
Methionine	2.01	1.83	1.39	1.27	1.27	—	1.27	1.12	0.96	1.10	0.92	1.05	0.88
Phenylalanine	2.77	2.60	2.38	2.22	2.50	—	2.08	2.01	1.98	1.86	1.80	1.80	1.74
Threonine	3.14	2.85	2.57	2.43	2.42	—	2.05	2.05	2.01	1.99	1.90	1.97	1.85
Tryptophan	0.45	0.47	0.46	0.47	0.38	—	0.41	0.43	0.45	0.42	0.45	0.38	0.37
Valine	3.04	3.40	2.99	2.58	2.99	0.90^5^	2.53	2.66	2.72	2.39	2.35	2.29	2.17
∑EAA^1^	30.28	30.26	26.50	24.35	27.42	—	22.09	22.00	21.68	21.03	20.27	20.68	19.75
Nonessential amino acid (%)													
Alanine	4.33	4.49	3.97	4.31	6.46	—	2.87	3.00	3.05	3.03	3.16	3.29	3.54
Aspartic acid	6.40	5.99	5.21	4.87	5.57	—	4.43	4.35	4.22	4.27	4.12	4.15	4.01
Cysteine	0.86	0.80	0.65	0.75	0.61	—	0.65	0.59	0.54	0.63	0.56	0.52	0.43
Glutamic acid	8.95	8.63	7.30	8.22	9.73	—	6.76	6.62	6.51	6.72	6.71	6.62	6.54
Glycine	3.83	4.68	4.25	6.05	11.94	—	2.55	2.99	3.18	3.61	4.65	4.12	5.61
Proline	2.82	3.06	2.84	3.94	7.38	—	2.05	2.21	2.31	2.69	3.35	2.79	3.64
Serine	2.94	2.49	2.36	2.46	2.90	—	1.77	1.65	1.56	1.68	1.51	1.56	1.42
Tyrosine	2.06	1.41	1.49	1.44	1.44	—	1.02	0.86	0.72	0.82	0.69	0.81	0.63
∑NEAA^2^	32.19	31.55	28.07	32.04	46.03	—	22.10	22.27	22.09	23.45	24.75	23.86	25.82

^1^∑EAA, total content of essential amino acid. ^2^∑NEAA, total content of nonessential amino acid. Arginine^3^, lysine^4^, and valine^5^ requirements were obtained from Rahimnejad and Lee [53], Forster and Ogata [54], and Rahimnejad and Lee [55], respectively.

**Table 3:** Fatty acid (% of total fatty acids) profiles of the protein sources and experimental diets.

Fatty acid (%)	Protein sources					Requirement	Experimental diets						
	FM	JMM	TBM	CBM	MM		Con	TBM25	TBM50	CBM25	CBM50	MM25	MM50
C12 : 0	0.11	0.05	0.10	0.08	0.16	—	0.15	0.09	0.09	0.08	0.05	0.06	0.07
C14 : 0	0.54	0.44	1.05	0.12	0.18	—	1.56	1.60	1.71	1.27	1.04	1.23	1.08
C16 : 0	23.34	21.42	27.36	28.12	28.08	—	15.18	15.62	16.84	16.05	17.76	15.58	16.66
C18 : 0	5.19	7.61	8.51	8.89	15.19	—	4.70	5.36	5.87	4.88	4.99	6.73	8.31
C20 : 0	0.14	0.15	0.24	0.05	0.03	—	0.25	0.21	0.20	0.22	0.21	0.21	0.22
C22 : 0	6.41	5.01	4.22	0.97	2.43	—	0.21	0.13	0.07	0.12	0.07	0.12	0.06
C24 : 0	2.56	3.31	1.26	0.04	0.03	—	0.16	0.15	0.10	0.10	0.08	0.15	0.10
∑SFA^1^	38.29	37.99	42.74	38.27	46.10	—	22.21	23.16	24.88	22.72	24.20	24.08	26.50
C14 : 1n-5	0.13	0.14	0.05	0.24	0.05	—	0.08	0.07	0.07	0.04	0.07	0.04	0.05
C15 : 1n-7	0.07	0.07	0.13	0.27	0.06	—	0.07	0.08	0.09	0.05	0.07	0.06	0.08
C16 : 1n-7	6.73	6.74	5.65	5.78	3.05	—	1.98	1.91	1.81	1.91	1.83	1.61	1.58
C17 : 1n-7	0.81	0.86	0.75	0.19	0.45	—	0.26	0.25	0.23	0.33	0.27	0.28	0.27
C18 : 1n-9	16.23	20.36	23.47	48.02	45.46	—	22.95	25.75	27.80	28.81	31.26	28.64	31.88
C20 : 1n-9	4.17	1.80	2.30	1.05	1.36	—	1.07	0.81	0.77	0.58	0.48	0.72	0.62
C22 : 1n-9	0.45	0.83	2.16	0.06	0.03	—	0.62	0.73	1.06	0.50	0.49	0.48	0.42
C24 : 1n-9	0.24	0.38	0.70	0.00	0.00	—	0.90	0.94	0.99	0.81	0.65	0.82	0.66
∑MUFA^2^	28.83	31.18	35.21	55.61	50.46	—	27.93	30.54	32.82	33.03	35.12	32.65	35.56
C18 : 2n-6	4.53	1.73	2.37	4.86	2.30	—	34.55	32.49	28.46	32.90	32.50	31.69	27.62
C18 : 3n-3	1.68	0.76	0.80	0.06	0.03	—	3.95	3.85	3.64	3.31	3.09	3.42	3.22
C18 : 3n-6	0.23	0.10	0.32	0.02	0.04	—	0.25	0.27	0.31	0.25	0.22	0.23	0.20
C20 : 2n-6	0.08	0.17	0.29	0.08	0.09	—	0.11	0.06	0.09	0.05	0.04	0.05	0.05
C20 : 3n-3	0.08	0.00	0.00	0.00	0.00	—	0.13	0.08	0.09	0.07	0.08	0.08	0.06
C20 : 3n-6	0.10	0.00	0.00	0.00	0.00	—	0.29	0.29	0.27	0.23	0.21	0.26	0.23
C20 : 4n-6	0.67	0.69	1.31	0.36	0.15	—	0.07	0.08	0.12	0.06	0.07	0.07	0.06
C20 : 5n-3	10.54	10.73	4.58	0.03	0.06	6.67^4^	3.87	3.20	2.96	2.81	1.90	2.73	1.97
C22 : 2n-6	0.23	0.33	0.26	0.04	0.11	—	0.16	0.19	0.19	0.16	0.14	0.17	0.13
C22 : 6n-3	13.30	15.40	10.48	0.01	0.02	3.33^4^	5.01	4.88	4.75	3.30	1.85	3.41	2.43
DHA:EPA ratio	1.26	1.44	2.29	0.33	0.33	—	1.29	1.53	1.60	1.17	0.97	1.25	1.23
∑n-3 HUFA^3^	23.92	26.13	15.06	0.04	0.08	—	9.01	8.16	7.80	6.18	4.26	6.22	4.46
Unknown	1.44	0.92	1.64	0.66	0.64	—	1.47	0.91	1.42	1.11	0.58	1.16	1.97

^1^∑SFA, total content of saturated fatty acid. ^2^∑MUFA, total content of monounsaturated fatty acid. ^3^∑n-3 HUFA, total content of n-3 highly unsaturated fatty acid. ^4^EPA and DHA requirements were obtained from Takeuchi et al. [57]'s study, in which one of both was not present in diets.

**Table 4:** Growth performance, feed availability, and biological indices of red sea bream (*Pagrus major*) fed the experimental diets for 8 weeks.

Experimental diets	Initial weight (g/fish)	Final weight (g/fish)	Survival (%)	Feed consumption (g/fish)	FE^1^	PER^2^	PR^3^ (%)	(K, g/cm^3^)	VSI^5^ (%)	HSI^6^ (%)
Con	11.9 ± 0.09	58.7 ± 0.72	90.0 ± 2.89	44.9 ± 0.70^ab^	1.06 ± 0.008^a^	1.99 ± 0.008^ab^	35.1 ± 1.48	2.09 ± 0.030	7.72 ± 0.036	2.25 ± 0.050
TBM25	11.7 ± 0.06	59.6 ± 0.30	91.7 ± 4.41	46.0 ± 0.29^a^	1.04 ± 0.010^ab^	2.02 ± 0.017^a^	35.9 ± 0.49	2.11 ± 0.027	7.24 ± 0.055	2.12 ± 0.029
TBM50	11.8 ± 0.10	61.0 ± 0.93	90.0 ± 2.89	47.3 ± 0.32^a^	1.05 ± 0.019^ab^	2.00 ± 0.032^ab^	36.4 ± 1.29	2.09 ± 0.043	7.41 ± 0.128	2.23 ± 0.155
CBM25	11.8 ± 0.06	54.3 ± 1.51	91.7 ± 1.67	40.9 ± 1.05^c^	1.04 ± 0.012^ab^	2.03 ± 0.019^a^	36.4 ± 1.15	2.08 ± 0.018	7.49 ± 0.226	2.25 ± 0.047
CBM50	11.8 ± 0.12	47.0 ± 0.78	98.3 ± 1.67	35.9 ± 1.01^d^	0.98 ± 0.006^c^	1.92 ± 0.014^b^	34.2 ± 1.30	2.06 ± 0.030	7.52 ± 0.198	2.08 ± 0.020
MM25	11.7 ± 0.12	59.2 ± 0.25	91.7 ± 1.67	45.2 ± 0.39^ab^	1.05 ± 0.003^ab^	2.04 ± 0.008^a^	36.6 ± 0.53	2.09 ± 0.053	7.39 ± 0.100	2.22 ± 0.133
MM50	11.9 ± 0.06	54.2 ± 1.10	93.3 ± 1.67	42.7 ± 0.45^bc^	0.99 ± 0.018^bc^	1.90 ± 0.031^b^	34.5 ± 0.39	2.05 ± 0.030	7.18 ± 0.420	2.30 ± 0.124
*P* value	—	—	*P* > 0.3	*P* < 0.0001	*P* < 0.004	*P* < 0.002	*P* > 0.3	*P* > 0.1	*P* > 0.3	*P* > 0.3
Main effect: substitution source										
TBM	—	—	—	46.6^A^	1.05	2.01	36.1	2.10	7.33	2.18
CBM	—	—	—	38.4^C^	1.01	1.97	35.3	2.07	7.51	2.17
MM	—	—	—	44.0^B^	1.02	1.98	35.6	2.07	7.29	2.26
Main effect: substitution level										
25%	—	—	—	44.1^A^	1.05^A^	2.03^A^	36.3	2.09	7.37	2.20
50%	—	—	—	42.0^B^	1.01^B^	1.94^B^	35.0	2.07	7.37	2.20
Two-way ANOVA	—	—	—	—	—	—	—	—	—	—
Substitution source	—	—	—	*P* < 0.0001	*P* > 0.06	*P* > 0.1	*P* > 0.6	*P* > 0.6	*P* > 0.5	*P* > 0.6
Substitution level	—	—	—	*P* < 0.003	*P* < 0.005	*P* < 0.0001	*P* > 0.1	*P* > 0.3	*P* > 0.9	*P* > 0.9
Interaction	—	—	—	*P* < 0.003	*P* < 0.04	*P* > 0.06	*P* > 0.3	*P* > 0.9	*P* > 0.6	*P* > 0.4

Values (means of triplicate ± SE) in the same column sharing the common superscript letter are not significantly different (*P* > 0.05). ^1^Feed efficiency (FE) = (Total final weight of fish (g) − total initial weight of fish (g) + total weight of dead fish (g))/total feed consumption of fish (g). ^2^Protein efficiency ratio (PER) = Weight gain of fish (g/fish)/protein consumption of fish (g/fish). ^3^Protein retention (PR, %) = Protein gain of fish (g/fish) × 100/protein consumption of fish (g/fish). ^4^Condition factor (K, g/cm^3^) = Body weight of fish (g) × 100/total length of fish (cm)^3^. ^5^Viscerosomatic index (VSI, %) = Viscera weight of fish (g) × 100/body weight of fish (g). ^6^ Hepatosomatic index (HSI, %) = Liver weight of fish (g) × 100 / body weight of fish (g).

**Table 5:** Plasma and serum parameters of red sea bream (*Pagrus major*) fed the experimental diets for 8 weeks.

	Experimental diets							*P* value	Main effect: substitution source			Main effect: substitution level		Two-way ANOVA		
	Con	TBM25	TBM50	CBM25	CBM50	MM25	MM50		TBM	CBM	MM	25%	50%	Substitution source	Substitution level	Interaction
Plasma parameters																
AST (U/L)	50.3 ± 1.54	55.3 ± 1.75	52.2 ± 1.68	53.6 ± 2.57	53.1 ± 2.18	50.9 ± 2.36	53.2 ± 2.82	*P* > 0.6	53.8	53.3	52.0	53.2	52.8	*P* > 0.6	*P* > 0.8	*P* > 0.4
ALT (U/L)	9.0 ± 0.62	8.3 ± 0.35	8.4 ± 0.29	8.2 ± 0.28	8.3 ± 0.38	9.1 ± 0.30	9.2 ± 0.32	*P* > 0.3	8.4	8.3	9.2	8.6	8.7	*P* > 0.06	*P* > 0.7	*P* > 0.9
ALP (U/L)	178.1 ± 8.54	167.0 ± 7.69	175.3 ± 7.35	174.9 ± 8.16	182.7 ± 6.41	179.2 ± 5.99	176.6 ± 5.55	*P* > 0.9	171.2	178.8	177.9	173.7	178.2	*P* > 0.6	*P* > 0.5	*P* > 0.7
T-BIL (mg/dL)	0.8 ± 0.05	0.9 ± 0.03	0.9 ± 0.03	0.9 ± 0.05	0.9 ± 0.04	0.9 ± 0.03	0.9 ± 0.06	*P* > 0.7	0.9	0.9	0.9	0.9	0.9	*P* > 0.6	*P* > 0.6	*P* > 0.6
T-CHO (mg/dL)	248.0 ± 7.30	254.2 ± 6.92	250.1 ± 5.05	264.0 ± 5.14	252.2 ± 5.25	246.4 ± 7.23	247.4 ± 7.98	*P* > 0.8	252.2	258.1	247.0	254.9	250.0	*P* > 0.4	*P* > 0.4	*P* > 0.7
TG (mg/dL)	390.0 ± 13.26	381.1 ± 7.05	383.8 ± 6.82	393.7 ± 9.51	381.3 ± 9.50	389.7 ± 10.05	389.0 ± 9.45	*P* > 0.9	382.4	387.5	389.3	388.1	384.7	*P* > 0.8	*P* > 0.7	*P* > 0.8
TP (g/dL)	4.7 ± 0.18	4.8 ± 0.16	4.8 ± 0.15	5.0 ± 0.17	5.1 ± 0.17	4.9 ± 0.18	5.1 ± 0.15	*P* > 0.8	4.8	5.1	5.0	4.9	5.0	*P* > 0.5	*P* > 0.6	*P* > 0.9
ALB (g/dL)	1.1 ± 0.05	1.1 ± 0.00	1.1 ± 0.05	1.1 ± 0.04	1.1 ± 0.03	1.1 ± 0.04	1.1 ± 0.04	*P* > 0.9	1.1	1.1	1.1	1.1	1.1	*P* > 0.6	*P* > 0.9	*P* > 0.6
Serum parameters																
Lysozyme activity (U/mL)	119.1 ± 8.54	105.0 ± 10.41	100.0 ± 15.00	100.0 ± 10.00	96.7 ± 7.26	125.0 ± 8.66	100.0 ± 5.77	*P* > 0.1	102.5	98.3	112.5	110.0	98.9	*P* > 0.3	*P* > 0.1	*P* > 0.5
SOD (%)	67.6 ± 0.56	67.7 ± 0.77	67.5 ± 0.68	67.1 ± 0.57	67.0 ± 0.51	67.2 ± 0.58	67.7 ± 0.43	*P* > 0.9	67.6	67.0	67.4	67.3	67.4	*P* > 0.7	*P* > 0.9	*P* > 0.9

Values (means of triplicate ± SE) in the same row sharing the same superscript letter are not significantly different (*P* > 0.05).

**Table 6:** Chemical composition (% of wet weight) of the whole body of red sea bream (*Pagrus major*) fed the experimental diets for 8 weeks.

Experimental diets	Moisture	Crude protein	Crude lipid	Ash
Con	64.2 ± 0.11	17.6 ± 0.63	10.5 ± 0.20	4.8 ± 0.25
TBM25	68.2 ± 2.04	17.8 ± 0.27	10.9 ± 0.29	5.2 ± 0.42
TBM50	66.3 ± 0.89	18.1 ± 0.38	10.2 ± 0.75	4.6 ± 0.25
CBM25	66.5 ± 0.78	17.9 ± 0.32	10.1 ± 0.98	4.8 ± 0.27
CBM50	67.1 ± 1.00	17.8 ± 0.54	9.2 ± 0.48	4.7 ± 0.43
MM25	65.7 ± 0.50	17.9 ± 0.26	10.9 ± 0.34	4.7 ± 0.27
MM50	67.9 ± 0.70	18.1 ± 0.07	10.5 ± 1.35	4.9 ± 0.45
*P* value	*P* > 0.1	*P* > 0.9	*P* > 0.7	*P* > 0.9
Main effect: substitution source				
TBM	67.3	18.0	10.6	4.9
CBM	66.8	17.9	9.7	4.8
MM	66.8	18.0	10.7	4.8
Main effect: substitution level				
25%	66.8	17.9	10.7	4.9
50%	67.1	18.0	10.0	4.8
Two-way ANOVA				
Substitution source	*P* > 0.8	*P* > 0.9	*P* > 0.4	*P* > 0.9
Substitution level	*P* > 0.7	*P* > 0.6	*P* > 0.3	*P* > 0.6
Interaction	*P* > 0.2	*P* > 0.8	*P* > 0.9	*P* > 0.5

Values (means of triplicate ± SE) in the same column sharing the same superscript letter are not significantly different (*P*  > 0.05).

**Table 7:** Amino acid profiles (% of wet weight) of the whole body of red sea bream (*Pagrus major*) fed the experimental diets for 8 weeks.

	Experimental diets							*P* value	Main effect: substitution source			Main effect: substitution level		Two-way ANOVA		
	Con	TBM25	TBM50	CBM25	CBM50	MM25	MM50		TBM	CBM	MM	25%	50%	Substitution source	Substitution level	Interaction
Arginine	1.04 ± 0.010	1.05 ± 0.006	1.02 ± 0.007	1.03 ± 0.006	1.02 ± 0.012	1.02 ± 0.009	1.03 ± 0.012	*P* > 0.3	1.04	1.03	1.02	1.03	1.03	*P* > 0.4	*P* > 0.3	*P* > 0.1
Histidine	0.40 ± 0.003	0.41 ± 0.010	0.40 ± 0.009	0.41 ± 0.010	0.40 ± 0.006	0.40 ± 0.006	0.41 ± 0.006	*P* > 0.9	0.41	0.40	0.41	0.41	0.40	*P* > 0.9	*P* > 0.8	*P* > 0.6
Isoleucine	0.66 ± 0.008	0.68 ± 0.009	0.68 ± 0.007	0.68 ± 0.013	0.67 ± 0.003	0.68 ± 0.012	0.68 ± 0.015	*P* > 0.7	0.68	0.67	0.69	0.68	0.68	*P* > 0.4	*P* > 0.7	*P* > 0.4
Leucine	1.20 ± 0.005	1.21 ± 0.009	1.20 ± 0.015	1.19 ± 0.013	1.17 ± 0.022	1.18 ± 0.012	1.19 ± 0.017	*P* > 0.5	1.21	1.18	1.18	1.20	1.18	*P* > 0.2	*P* > 0.2	*P* > 0.8
Lysine	1.37 ± 0.013	1.37 ± 0.017	1.37 ± 0.015	1.39 ± 0.016	1.36 ± 0.030	1.38 ± 0.020	1.37 ± 0.009	*P* > 0.8	1.37	1.38	1.38	1.38	1.37	*P* > 0.8	*P* > 0.4	*P* > 0.5
Methionine	0.49 ± 0.020	0.48 ± 0.027	0.49 ± 0.023	0.48 ± 0.023	0.51 ± 0.015	0.51 ± 0.015	0.48 ± 0.029	*P* > 0.8	0.49	0.49	0.51	0.49	0.50	*P* > 0.6	*P* > 0.4	*P* > 0.6
Phenylalanine	0.62 ± 0.010	0.64 ± 0.009	0.63 ± 0.003	0.65 ± 0.003	0.63 ± 0.007	0.64 ± 0.007	0.65 ± 0.027	*P* > 0.5	0.64	0.64	0.65	0.64	0.64	*P* > 0.5	*P* > 0.6	*P* > 0.1
Threonine	0.72 ± 0.010	0.71 ± 0.012	0.69 ± 0.003	0.71 ± 0.009	0.70 ± 0.009	0.69 ± 0.009	0.68 ± 0.009	*P* > 0.1	0.70	0.70	0.69	0.70	0.69	*P* > 0.3	*P* > 0.08	*P* > 0.7
Tryptophan	0.10 ± 0.000	0.10 ± 0.003	0.11 ± 0.006	0.10 ± 0.006	0.10 ± 0.007	0.10 ± 0.007	0.11 ± 0.008	*P* > 0.5	0.10	0.10	0.10	0.10	0.11	*P* > 0.9	*P* > 0.1	*P* > 0.4
Valine	0.75 ± 0.010	0.76 ± 0.012	0.76 ± 0.007	0.77 ± 0.019	0.76 ± 0.009	0.75 ± 0.012	0.77 ± 0.006	*P* > 0.8	0.76	0.76	0.76	0.76	0.76	*P* > 0.9	*P* > 0.7	*P* > 0.5
Alanine	1.14 ± 0.016	1.15 ± 0.003	1.15 ± 0.015	1.14 ± 0.018	1.15 ± 0.009	1.14 ± 0.023	1.13 ± 0.015	*P* > 0.9	1.15	1.14	1.13	1.15	1.14	*P* > 0.6	*P* > 0.6	*P* > 0.7
Aspartic acid	1.52 ± 0.010	1.53 ± 0.015	1.52 ± 0.018	1.52 ± 0.018	1.49 ± 0.015	1.51 ± 0.010	1.51 ± 0.019	*P* > 0.7	1.53	1.51	1.52	1.52	1.51	*P* > 0.5	*P* > 0.5	*P* > 0.5
Cysteine	0.21 ± 0.007	0.20 ± 0.009	0.22 ± 0.013	0.22 ± 0.000	0.22 ± 0.013	0.22 ± 0.007	0.21 ± 0.010	*P* > 0.6	0.21	0.22	0.22	0.21	0.22	*P* > 0.4	*P* > 0.5	*P* > 0.4
Glutamic acid	2.22 ± 0.013	2.25 ± 0.012	2.22 ± 0.017	2.23 ± 0.010	2.20 ± 0.032	2.22 ± 0.020	2.25 ± 0.015	*P* > 0.4	2.24	2.22	2.24	2.23	2.23	*P* > 0.5	*P* > 0.8	*P* > 0.1
Glycine	1.41 ± 0.034	1.39 ± 0.023	1.40 ± 0.032	1.37 ± 0.021	1.40 ± 0.036	1.35 ± 0.036	1.34 ± 0.037	*P* > 0.7	1.40	1.39	1.34	1.37	1.38	*P* > 0.3	*P* > 0.7	*P* > 0.7
Proline	0.83 ± 0.019	0.82 ± 0.015	0.82 ± 0.006	0.67 ± 0.013	0.81 ± 0.019	0.80 ± 0.006	0.81 ± 0.012	*P* > 0.9	0.82	0.82	0.81	0.81	0.81	*P* > 0.5	*P* > 0.8	*P* > 0.8
Serine	0.67 ± 0.013	0.67 ± 0.015	0.68 ± 0.017	0.36 ± 0.012	0.66 ± 0.015	0.66 ± 0.019	0.67 ± 0.018	*P* > 0.8	0.68	0.66	0.66	0.67	0.67	*P* > 0.6	*P* > 0.8	*P* > 0.7
Tyrosine	0.37 ± 0.016	0.37 ± 0.023	0.35 ± 0.015	0.36 ± 0.013	0.37 ± 0.018	0.35 ± 0.012	0.35 ± 0.023	*P* > 0.8	0.36	0.37	0.35	0.36	0.36	*P* > 0.7	*P* > 0.7	*P* > 0.8

Values (means of triplicate ± SE) in the same row sharing the same superscript letter are not significantly different (*P* > 0.05).

**Table 8:** Fatty acid profiles (% of wet weight) of the whole body of red sea bream (*Pagrus major*) fed the experimental diets for 8 weeks.

	Experimental diets							*P* value	Main effect: substitution source			Main effect: substitution level		Two-way ANOVA		
	Con	TBM25	TBM50	CBM25	CBM50	MM25	MM50		TBM	CBM	MM	25%	50%	Substitution source	Substitution level	Interaction
C12 : 0	0.06 ± 0.009	0.05 ± 0.007	0.06 ± 0.009	0.07 ± 0.013	0.05 ± 0.013	0.05 ± 0.012	0.06 ± 0.007	*P* > 0.8	0.06	0.06	0.06	0.06	0.06	*P* > 0.8	*P* > 0.8	*P* > 0.3
C14 : 0	1.45 ± 0.052	1.42 ± 0.010	1.42 ± 0.032	1.42 ± 0.055	1.39 ± 0.069	1.42 ± 0.018	1.48 ± 0.093	*P* > 0.9	1.42	1.40	1.45	1.42	1.43	*P* > 0.7	*P* > 0.8	*P* > 0.7
C16 : 0	15.85 ± 0.062	15.72 ± 0.323	15.65 ± 0.139	16.00 ± 0.141	15.86 ± 0.269	15.99 ± 0.261	15.83 ± 0.284	*P* > 0.9	15.69	15.93	15.91	15.90	15.78	*P* > 0.5	*P* > 0.5	*P* > 0.9
C18 : 0	6.61 ± 0.236	6.55 ± 0.112	6.68 ± 0.125	6.56 ± 0.142	6.54 ± 0.086	6.76 ± 0.151	6.53 ± 0.322	*P* > 0.9	6.62	6.55	6.65	6.62	6.58	*P* > 0.8	*P* > 0.7	*P* > 0.6
C20 : 0	0.21 ± 0.009	0.18 ± 0.017	0.20 ± 0.020	0.21 ± 0.012	0.20 ± 0.003	0.19 ± 0.015	0.19 ± 0.025	*P* > 0.8	0.19	0.21	0.19	0.19	0.20	*P* > 0.6	*P* > 0.8	*P* > 0.8
C22 : 0	0.54 ± 0.022	0.52 ± 0.018	0.54 ± 0.009	0.51 ± 0.010	0.51 ± 0.006	0.53 ± 0.010	0.51 ± 0.034	*P* > 0.7	0.53	0.51	0.52	0.52	0.52	*P* > 0.5	*P* > 0.9	*P* > 0.6
C24 : 0	0.22 ± 0.035	0.22 ± 0.017	0.21 ± 0.034	0.24 ± 0.035	0.27 ± 0.031	0.27 ± 0.015	0.22 ± 0.026	*P* > 0.6	0.22	0.26	0.25	0.24	0.24	*P* > 0.3	*P* > 0.7	*P* > 0.4
∑SFA^1^	24.90 ± 0.311	24.62 ± 0.360	24.70 ± 0.170	24.94 ± 0.333	24.77 ± 0.277	25.16 ± 0.250	24.77 ± 0.387	*P* > 0.8	24.66	24.85	24.97	24.91	24.75	*P* > 0.6	*P* > 0.5	*P* > 0.7
C14 : 1n-5	0.04 ± 0.003	0.05 ± 0.009	0.05 ± 0.009	0.05 ± 0.003	0.05 ± 0.009	0.04 ± 0.007	0.05 ± 0.006	*P* > 0.9	0.05	0.05	0.05	0.05	0.05	*P* > 0.8	*P* > 0.9	*P* > 0.6
C15 : 1n-7	0.04 ± 0.003	0.04 ± 0.003	0.04 ± 0.003	0.04 ± 0.000	0.04 ± 0.007	0.04 ± 0.007	0.04 ± 0.007	*P* > 0.8	0.04	0.04	0.04	0.04	0.04	*P* > 0.8	*P* > 0.4	*P* > 0.8
C16 : 1n-7	2.57 ± 0.164	2.59 ± 0.059	2.55 ± 0.111	2.52 ± 0.111	2.60 ± 0.142	2.52 ± 0.131	2.46 ± 0.046	*P* > 0.9	2.57	2.56	2.49	2.54	2.54	*P* > 0.7	*P* > 0.9	*P* > 0.7
C17 : 1n-7	0.34 ± 0.015	0.34 ± 0.009	0.34 ± 0.006	0.36 ± 0.019	0.39 ± 0.029	0.35 ± 0.015	0.37 ± 0.003	*P* > 0.2	0.34	0.38	0.36	0.35	0.37	*P* > 0.07	*P* > 0.1	*P* > 0.7
C18 : 1n-9	26.37 ± 0.113^b^	28.06 ± 0.351^ab^	28.79 ± 0.359^a^	28.28 ± 0.215^a^	29.40 ± 0.647^a^	27.80 ± 0.327^ab^	29.01 ± 0.269^a^	*P* < 0.002	28.43	28.84	28.41	28.05^B^	29.07^A^	*P* > 0.4	*P* < 0.01	*P* > 0.8
C20 : 1n-9	0.96 ± 0.052	0.95 ± 0.028	0.94 ± 0.073	0.98 ± 0.057	1.03 ± 0.084	0.95 ± 0.078	0.99 ± 0.050	*P* > 0.9	0.95	1.00	0.97	0.96	0.97	*P* > 0.6	*P* > 0.6	*P* > 0.8
C22 : 1n-9	0.44 ± 0.032^b^	0.60 ± 0.009^ab^	0.72 ± 0.030^a^	0.43 ± 0.059^b^	0.44 ± 0.012^b^	0.45 ± 0.017^b^	0.53 ± 0.053^b^	*P* < 0.0001	0.66^A^	0.44^B^	0.49^B^	0.50^B^	0.56^A^	*P* < 0.0001	*P* < 0.04	*P* > 0.3
C24 : 1n-9	1.18 ± 0.039	1.20 ± 0.017	1.12 ± 0.083	1.15 ± 0.046	1.16 ± 0.021	1.20 ± 0.020	1.17 ± 0.015	*P* > 0.8	1.16	1.16	1.19	1.18	1.15	*P* > 0.7	*P* > 0.3	P > 0.5
∑MUFA^2^	31.94 ± 0.279^b^	33.83 ± 0.315^ab^	34.55 ± 0.236^a^	33.82 ± 0.316^ab^	35.11 ± 0.865^a^	33.35 ± 0.157^ab^	34.63 ± 0.328^a^	*P* < 0.003	34.19	34.47	33.99	33.67^B^	34.76^A^	*P* > 0.5	*P* < 0.01	*P* > 0.7
C18 : 2n-6	31.07 ± 0.269^a^	30.18 ± 0.430^ab^	29.91 ± 0.164^ab^	30.26 ± 0.657^ab^	28.58 ± 0.344^b^	30.02 ± 0.172^ab^	29.78 ± 0.460^ab^	*P* < 0.03	30.05	29.42	29.90	30.15^A^	29.42^B^	*P* > 0.3	*P* < 0.05	*P* > 0.1
C18 : 3n-3	2.86 ± 0.098	2.92 ± 0.109	2.85 ± 0.044	2.83 ± 0.084	2.88 ± 0.101	2.81 ± 0.047	2.90 ± 0.095	*P* > 0.9	2.89	2.86	2.85	2.85	2.88	*P* > 0.8	*P* > 0.7	*P* > 0.6
C18 : 3n-6	0.22 ± 0.023	0.24 ± 0.018	0.22 ± 0.012	0.21 ± 0.015	0.22 ± 0.024	0.22 ± 0.030	0.22 ± 0.015	*P* > 0.9	0.23	0.21	0.22	0.22	0.22	*P* > 0.6	*P* > 0.8	*P* > 0.7
C20 : 2n-6	0.12 ± 0.017	0.11 ± 0.022	0.11 ± 0.012	0.10 ± 0.003	0.12 ± 0.017	0.11 ± 0.012	0.09 ± 0.006	*P* > 0.7	0.11	0.11	0.10	0.11	0.11	*P* > 0.7	*P* > 0.9	*P* > 0.3
C20 : 3n-3	0.23 ± 0.023	0.24 ± 0.023	0.25 ± 0.006	0.25 ± 0.015	0.26 ± 0.006	0.22 ± 0.006	0.21 ± 0.012	*P* > 0.3	0.25^AB^	0.25^A^	0.21^B^	0.24	0.24	*P* < 0.04	*P* > 0.6	*P* > 0.7
C20 : 3n-6	0.14 ± 0.015	0.14 ± 0.010	0.15 ± 0.009	0.15 ± 0.012	0.15 ± 0.009	0.17 ± 0.012	0.14 ± 0.030	*P* > 0.8	0.14	0.15	0.16	0.15	0.15	*P* > 0.7	*P* > 0.7	*P* > 0.5
C20 : 4n-6	0.22 ± 0.017	0.23 ± 0.021	0.24 ± 0.022	0.25 ± 0.009	0.22 ± 0.023	0.23 ± 0.017	0.23 ± 0.015	*P* > 0.9	0.24	0.24	0.23	0.24	0.23	*P* > 0.8	*P* > 0.7	*P* > 0.6
C20 : 5n-3	2.62 ± 0.038^a^	2.51 ± 0.073^a^	2.24 ± 0.039^b^	2.15 ± 0.037^b^	1.86 ± 0.049^c^	2.19 ± 0.034^b^	1.79 ± 0.017^c^	*P* < 0.0001	2.38^A^	2.00^B^	1.99^B^	2.28^A^	1.96^B^	*P* < 0.0001	*P* < 0.0001	*P* > 0.3
C22 : 2n-6	0.24 ± 0.012	0.26 ± 0.010	0.25 ± 0.012	0.26 ± 0.015	0.27 ± 0.019	0.24 ± 0.012	0.25 ± 0.003	*P* > 0.6	0.26	0.26	0.25	0.25	0.26	*P* > 0.4	*P* > 0.5	*P* > 0.6
C22 : 6n-3	4.66 ± 0.127^a^	4.32 ± 0.035^b^	4.16 ± 0.031^bc^	3.90 ± 0.067^c^	3.45 ± 0.035^d^	4.01 ± 0.070^bc^	3.52 ± 0.061^d^	*P* < 0.0001	4.24^A^	3.68^B^	3.77^B^	4.07^A^	3.71^B^	*P* < 0.0001	*P* < 0.0001	*P* < 0.02
∑n-3 HUFA^3^	7.51 ± 0.181^a^	7.07 ± 0.030^b^	6.65 ± 0.055^bc^	6.29 ± 0.087^c^	5.57 ± 0.064^d^	6.42 ± 0.047^c^	5.53 ± 0.061^d^	*P* < 0.0001	6.86^A^	5.93^B^	5.97^B^	6.60^A^	5.92^B^	*P* < 0.0001	*P* < 0.0001	*P* < 0.01
Unknown	0.78 ± 0.055	0.39 ± 0.057	0.35 ± 0.076	0.89 ± 0.325	2.11 ± 0.425	1.28 ± 0.247	1.47 ± 0.552	—	—	—	—	—	—	—	—	—

Values (means of triplicate ± SE) in the same row sharing the same superscript letter are not significantly different (*P* > 0.05). ^1^∑SFA, total content of saturated fatty acid. ^2^∑MUFA, total content of monounsaturated fatty acid. ^3^∑n-3 HUFA, total content of n-3 highly unsaturated fatty acid.

**Table 9:** Diet price (USD/kg) and economic parameters of the experimental diets for red sea bream (*Pagrus major*).

Experimental diets	Diet price (USD/kg)	ECR^1^ (USD/kg)	EPI^2^ (USD/fish)
Con	1.73	1.32 ± 0.007^a^	1.11 ± 0.013^ab^
TBM25	1.70	1.32 ± 0.009^ab^	1.13 ± 0.006^a^
TBM50	1.60	1.24 ± 0.014^cd^	1.16 ± 0.019^a^
CBM25	1.63	1.23 ± 0.003^cd^	1.03 ± 0.029^b^
CBM50	1.46	1.11 ± 0.013^e^	0.90 ± 0.014^c^
MM25	1.66	1.27 ± 0.006^bc^	1.12 ± 0.005^a^
MM50	1.52	1.20 ± 0.012^d^	1.04 ± 0.022^b^
*P* value		*P* < 0.0001	*P* < 0.0001
Main effect: substitution source			
TBM		1.28^A^	1.15^A^
CBM		1.17^C^	0.97^C^
MM		1.24^B^	1.08^B^
Main effect: substitution level			
25%		1.27^A^	1.10^A^
50%		1.19^B^	1.03^B^
Substitution source		*P* < 0.0001	*P* < 0.0001
Substitution level		*P* < 0.0001	*P* < 0.002
Interaction		*P* > 0.1	*P* < 0.002

Values (means of triplicate ± SE) in the same column sharing the common superscript letter are not significantly different (*P* > 0.05). ^1^Economic conversion ratio (ECR, USD/kg) = Feed consumption (kg/fish)/weight gain (kg/fish) × diet price (USD/kg). ^2^Economic profit index (EPI, USD/fish) = (Final weight (kg/fish) × selling price of fish (USD/kg))− [feed consumption (kg/fish) × diet price (USD/kg)].

## Data Availability

Data will be available from the corresponding authors by reasonable request.
